# Supplementary material for: Resolving the full spectrum of human genome variation using Linked-Reads
Source: Genome Res. 2019 Apr;29(4):635–45. doi: 10.1101/gr.234443.118 (PMC6442396; doi:10.1101/gr.234443.118)
Supplement: Supplemental Material [file supp_gr.234443.118_Supplemental_longranger-2.2.zip › longranger-2.2.tar.bz2!/lr2.2-public/lib/stan/cmdstan/stan/doc/api/html/agrad_2fwd_2matrix_8hpp_source.html]

Stan: src/stan/agrad/fwd/matrix.hpp Source File


|  |  |
| --- | --- |
|  | Stan  2.5.0  probability, sampling & optimization |


- Main Page
- Related Pages
- Namespaces
- Classes
- Files

- File List
- File Members

All Classes Namespaces Files Functions Variables Typedefs Enumerations Enumerator Friends Macros Pages

- src
- stan
- agrad
- fwd

matrix.hpp

Go to the documentation of this file.

1 #ifndef STAN\_\_AGRAD\_\_FWD\_\_MATRIX\_HPP

2 #define STAN\_\_AGRAD\_\_FWD\_\_MATRIX\_HPP

3

4 #include <stan/agrad/fwd/matrix/columns\_dot\_product.hpp>

5 #include <stan/agrad/fwd/matrix/columns\_dot\_self.hpp>

6 #include <stan/agrad/fwd/matrix/crossprod.hpp>

7 #include <stan/agrad/fwd/matrix/determinant.hpp>

8 #include <stan/agrad/fwd/matrix/divide.hpp>

9 #include <stan/agrad/fwd/matrix/dot\_product.hpp>

10 #include <stan/agrad/fwd/matrix/dot\_self.hpp>

11 #include <stan/agrad/fwd/matrix/inverse.hpp>

12 #include <stan/agrad/fwd/matrix/log\_determinant.hpp>

13 #include <stan/agrad/fwd/matrix/log\_softmax.hpp>

14 #include <stan/agrad/fwd/matrix/log\_sum\_exp.hpp>

15 #include <stan/agrad/fwd/matrix/mdivide\_left.hpp>

16 #include <stan/agrad/fwd/matrix/mdivide\_left\_ldlt.hpp>

17 #include <stan/agrad/fwd/matrix/mdivide\_left\_tri\_low.hpp>

18 #include <stan/agrad/fwd/matrix/mdivide\_right.hpp>

19 #include <stan/agrad/fwd/matrix/mdivide\_right\_tri\_low.hpp>

20 #include <stan/agrad/fwd/matrix/multiply.hpp>

21 #include <stan/agrad/fwd/matrix/multiply\_lower\_tri\_self\_transpose.hpp>

22 #include <stan/agrad/fwd/matrix/qr\_Q.hpp>

23 #include <stan/agrad/fwd/matrix/qr\_R.hpp>

24 #include <stan/agrad/fwd/matrix/rows\_dot\_product.hpp>

25 #include <stan/agrad/fwd/matrix/rows\_dot\_self.hpp>

26 #include <stan/agrad/fwd/matrix/softmax.hpp>

27 #include <stan/agrad/fwd/matrix/sort.hpp>

28 #include <stan/agrad/fwd/matrix/sum.hpp>

29 #include <stan/agrad/fwd/matrix/tcrossprod.hpp>

30 #include <stan/agrad/fwd/matrix/trace\_quad\_form.hpp>

31 #include <stan/agrad/fwd/matrix/trace\_gen\_quad\_form.hpp>

32 #include <stan/agrad/fwd/matrix/to\_fvar.hpp>

33 #include <stan/agrad/fwd/matrix/typedefs.hpp>

34

35 #endif

sum.hpp

log\_sum\_exp.hpp

inverse.hpp

mdivide\_left\_ldlt.hpp

typedefs.hpp

dot\_self.hpp

rows\_dot\_self.hpp

mdivide\_right\_tri\_low.hpp

divide.hpp

multiply\_lower\_tri\_self\_transpose.hpp

qr\_R.hpp

dot\_product.hpp

trace\_quad\_form.hpp

log\_determinant.hpp

rows\_dot\_product.hpp

log\_softmax.hpp

determinant.hpp

columns\_dot\_self.hpp

mdivide\_right.hpp

tcrossprod.hpp

mdivide\_left\_tri\_low.hpp

crossprod.hpp

to\_fvar.hpp

columns\_dot\_product.hpp

softmax.hpp

trace\_gen\_quad\_form.hpp

qr\_Q.hpp

multiply.hpp

mdivide\_left.hpp

sort.hpp


---

[ Stan Home Page ]

*© 2011–2014,
Stan Development Team.*
